# Supplementary material for: Efficient Genomic Interval Queries Using Augmented Range Trees
Source: Sci Rep. 2019 Mar 25;9:5059. doi: 10.1038/s41598-019-41451-3 (PMC6434014; doi:10.1038/s41598-019-41451-3)
Supplement: Supplementary file 1 — Supplementary Information: Efficient Genomic Interval Queries Using Augmented Range Trees [file 41598_2019_41451_MOESM1_ESM.pdf]

Supplementary Information:

Efficient Genomic Interval Queries Using Augmented Range Trees

Chengsheng Mao[1], Alal Eran[2, 3], Yuan Luo[1, *]

[1]Department of Preventive Medicine, Feinberg School of Medicine, Northwestern University, Chicago, IL, USA;

[2]Department of Biomedical Informatics, Harvard Medical School, Boston, MA, USA;

[3]Department of Life Sciences, Ben Gurion University of the Negev, Israel;

*Corresponding author: yuan.luo@northwestern.edu

S1. Test findOverlaps() code

```
library(IRanges)
print(R.Version()$version.string)
readAsRanges<-function(filename){
    cat(sprintf('reading file: %s\n',filename))
    itvs = read.table(filename)
    rg = IRanges(start=itvs$V1, end=itvs$V2)
    return(rg)
}

cat(paste('chromosome','any','within','start','end','equal'),
file="timeout_gr.txt",sep="\n",append=TRUE)
for (chr in c(1:22, 'X')) {
    datafile=sprintf('/home/shared_data/ENCODE/unqitv/chr%s.txt',chr)

queryfile=sprintf('/share/fsmresfiles/gnomad/gnomADr2.0.1/vcf/genomes
/genomes.r2.0.1.sites.21.csv',chr)
    gr1 = readAsRanges(datafile)
    gr1 = NCList(gr1)
    gr2 = readAsRanges(queryfile)
    gc()

    cat(sprintf('find any overlaps\n'))
    timestart<-Sys.time()
    findOverlaps(gr1, gr2,type='any')
    timeend<-Sys.time()
    t_any = as.double(timeend-timestart, units = "secs" )
    gc()

    cat(sprintf('find within overlaps\n'))
    timestart<-Sys.time()
    findOverlaps(gr1, gr2,type='within')  # 4 relations: d, s, f, e
    timeend<-Sys.time()
    t_within = as.double(timeend-timestart, units = "secs" )
    gc()

    cat(sprintf('find start overlaps\n'))
    timestart<-Sys.time()
    findOverlaps(gr1, gr2,type='start')  # 3 relations: s, si, e
    timeend<-Sys.time()
    t_start = as.double(timeend-timestart, units = "secs" )
    gc()

    cat(sprintf('find end overlaps\n'))
    timestart<-Sys.time()
    findOverlaps(gr1, gr2,type='end')  # 3 relations: f, fi, e
```

```
    timeend<-Sys.time()
    t_end = as.double(timeend-timestart, units = "secs" )

    cat(sprintf('find equal overlaps\n'))
    timestart<-Sys.time()
    findOverlaps(gr1, gr2,type='equal')  # 1 relations: e
    timeend<-Sys.time()
    t_equal = as.double(timeend-timestart, units = "secs" )
    gc()

    cat(paste(chr, t_any,t_within,t_start,t_end,t_equal),
file="timeout_gr.txt",sep="\n",append=TRUE)
}
```

S2. Interval counts in each chromosome

Chromosome	ENCODE intervals	gnomAD intervals
chr1	130,755,065	18,738,359
chr2	109,795,844	20,204,527
chr3	85,226,468	16,486,570
chr4	62,323,066	16,097,457
chr5	80,137,408	15,061,956
chr6	88,374,652	14,016,946
chr7	75,486,885	13,543,375
chr8	61,585,063	12,963,423
chr9	49,505,731	10,561,010
chr10	63,396,858	11,139,488
chr11	61,169,255	11,380,124
chr12	66,165,580	10,975,733
chr13	32,024,993	8,030,807
chr14	40,938,091	7,574,648
chr15	42,659,615	7,095,201
chr16	46,250,642	7,847,535
chr17	56,256,538	6,717,017
chr18	28,192,149	6,353,949
chr19	45,208,927	5,400,183
chr20	37,954,390	5,063,026
chr21	17,283,366	3,185,805
chr22	22,644,544	3,245,659
chrX	36,790,451	9,373,753
total	1,340,125,581	241,056,551

S3. The building time (in seconds) of the three tree structures on ENCODE genomic intervals for each chromosome.

Chromosome	RTFC	2D-RT	IT
chr1	749.23	1259.65	**415.87**
chr2	606.40	1039.65	**400.09**
chr3	456.99	680.08	**303.82**
chr4	315.89	541.14	**168.16**
chr5	426.93	710.28	**246.75**
chr6	480.34	825.22	**267.85**
chr7	401.38	580.27	**221.49**
chr8	315.30	544.01	**173.46**
chr9	249.58	417.13	**135.34**
chr10	328.04	554.97	**174.88**
chr11	314.78	532.39	**173.40**
chr12	345.88	585.10	**194.14**
chr13	147.71	249.00	**77.88**
chr14	199.04	336.07	**107.22**
chr15	211.76	351.60	**113.49**
chr16	231.40	379.06	**127.23**
chr17	294.13	484.06	**145.69**
chr18	131.49	218.94	**63.52**
chr19	229.74	371.54	**120.93**
chr20	187.66	314.78	**99.71**
chr21	75.40	126.74	**37.68**
chr22	105.05	173.46	**50.64**
chrX	167.48	294.04	**85.03**
total	6971.58	11569.15	**3904.27**

RTFC=range tree with fractional cascading; 2D-RT=basic 2-dimensional range tree; IT=interval tree.
